# General practitioners and sickness certification for injury in Australia

**DOI:** 10.1186/s12875-015-0307-9

**Published:** 2015-08-15

**Authors:** Danielle Mazza, Bianca Brijnath, Nabita Singh, Agnieszka Kosny, Rasa Ruseckaite, Alex Collie

**Affiliations:** Department of General Practice, School of Primary Care, Faculty of Medicine Nursing and Health Sciences, Monash University, Building 1, 270 Ferntree Gully Rd, Notting Hill, VIC 3168 Melbourne, Australia; Institute for Work and Health, University of Toronto, Toronto, Canada; Department of Epidemiology and Preventive Medicine, School of Public Health and Preventive Medicine, Faculty of Medicine Nursing and Health Sciences, Monash University, Melbourne, Australia; Institute for Safety Compensation and Recovery Research, Monash University, Melbourne, Australia

**Keywords:** Australia, Certification, General practitioners, Injury, Return to work

## Abstract

**Background:**

Strong evidence supports an early return to work after injury as a way to improve recovery. In Australia, General Practitioners (GPs) see about 96 % of injured workers, making them the main gatekeepers to workers’ entitlements. Most people with compensable injuries in Australia are certified as “unfit to work” by their GP, with a minority of patients certified for modified work duties. The reasons for this apparent dissonance between evidence and practice remain unexplored. Little is known about the factors that influence GP sickness certification behaviour in Australia. The aim of this study is to describe the factors influencing Australian GPs certification practice through qualitative interviews with four key stakeholders.

**Methods:**

From September to December 2012, 93 semi-structured interviews were undertaken in Melbourne, Australia. Participants included GPs, injured workers, employers and compensation agents. Data were thematically analysed.

**Results:**

Five themes describing factors influencing GP certification were identified: 1. Divergent stakeholder views about the GP’s role in facilitating return to work; 2. Communication between the four stakeholder groups; 3. Conflict between the stakeholder groups; 4. Allegations of GPs and injured workers misusing the compensation system and 5. The layout and content of the sickness certificate itself.

**Conclusion:**

By exploring GP certification practice from the perspectives of four key stakeholders, this study suggests that certification is an administrative and clinical task underpinned by a host of social and systemic factors. The findings highlight opportunities such as practice guideline development and improvements to the sickness certificate itself that may be targeted to improve GP sickness certification behaviour and return to work outcomes in an Australian context.

**Electronic supplementary material:**

The online version of this article (doi:10.1186/s12875-015-0307-9) contains supplementary material, which is available to authorized users.

## Background

In Australia, general practitioners (GPs) see about 96 % of injured workers, and are considered the main gatekeepers to workers entitlements [1]. They medically assess injured patients’ capacities and provide advice regarding the medical and care treatments necessary for recovery. Through certification, GPs recommend periods of time off work, make decisions that affect the liabilities of compensation agencies and provide case managers information for them to make inferences about injury causality (work or transport related). GPs therefore play a critical role in recovery and return to work (RTW) processes.

There is a strong emerging evidence-base on the benefits of early RTW after injury as a way to improve recovery [2, 3]. Studies show that delayed periods of time away from work can lead to or exacerbate mental health problems [4, 5], increase social isolation, reduce income and increase the burden on healthcare and compensation systems [6]. Alongside, there is also now strong evidence about the links between good health and meaningful work [6–8].

In Australia, there appears to be a dissonance between this evidence of the health benefits of RTW and GP certification practice. Recent analyses of Australian GP sickness certification reveal that about three-quarters (75.7 %) of all GP consultations related to work can be reimbursed through workers’ compensation [9, 10]. Only 22.7 % of initial certificates issued by GPs recommend ‘modified or alternate duties’ [11]. Over 70 % classify patients as ‘unfit for work’. For initial certificates relating to mental health conditions, up to 94 % recommend patients as ‘unfit for work’ [11]. This trend has been consistent for more than seven years [11, 12]. The reasons for this apparent dissonance between evidence and practice remains unknown. To date little is known about the factors that may influence GP sickness certification in an Australian context. Thus the key research question informing this article is: What factors influence GPs sickness certification practices for compensable injury in Australia?

Insights into the potential factors influencing GP sickness certification may be gained from previous studies in other contexts. In the UK, one in ten adults receives a sickness certificate [13] and research shows that GP certification practice often contradicts guidelines put forth by the UK Government’s Department of Work and Pensions [14, 15]. Reasons suggested for these high rates of certification include: low awareness of the guidelines, lack of training in sickness certification, experiencing conflict with patients and other stakeholders and GP’s having a different understanding of their role compared with other stakeholders (e.g. compensation authorities) [15]. Similar findings have been reported in the Netherlands [16], Switzerland [17], Norway [18] and Sweden [19–21].

The Australian environment is substantially different to that of the UK and Europe, with a different societal architecture in place, and different administrative and governance arrangements determining the operations of Australian injury compensation and healthcare systems. To gain insight into GP certification practice in the Australian context, the aim of this study is to describe the factors influencing Australian GPs certification practice through qualitative interviews with four key stakeholders.

## Methods

The data for this paper comes from a large qualitative study, which utilised a descriptive approach [22, 23], to explore four stakeholders – GPs, injured workers, employers and compensation agents – views on the barriers and enablers they encountered in facilitating RTW of injured workers. In this paper we specifically focus on stakeholders’ views about GPs certification practice.

### Study context

Data were collected in Melbourne, Victoria where state legislation mandates that claims for compensable injury must either be lodged at WorkSafe Victoria (WSV) – for work-related injuries – or the Transport Accident Commission (TAC) – for vehicle-related injuries. Comparable schemes exist in other states and territories. One purpose of these statutory compensation bodies is to underwrite an injured persons’ access to GPs and other health providers for treatment related to their compensable accident or injury. Medicare is the federally-funded health insurance scheme for all Australian citizens and Australian permanent residents; however, in the event a person sustains a work or transport related injury or illness, organisations such as WSV and TAC cover the costs associated with the injury rather than Medicare. Reimbursement of GP services for compensable injury is at a lower rate compared to Medicare; therefore there is no financial incentive for GPs to treat more compensable injury patients than other patients.

To commence a claim, a complete injury claim form and a complete initial certificate of capacity by a medical practitioner must be lodged by the injured worker’s employer to WSV and by the injured person to the TAC (depending on the injury) [24, 25]. Upon the claim’s assessment and acceptance, compensation payments are issued for lost income, medical and other expenses. The expectation is that the injured person will make a safe, appropriate and timely RTW in due course and that they and their compensation agent, employer and medical practitioner will jointly coordinate the RTW process [24, 25].

### Participants and sampling

A detailed description of the methods has been previously published [5]. To summarise: between September and December 2012, 93 semi-structured, face to face interviews were conducted with 25 GPs, 17 injured workers, 25 employers and 26 compensation agents. To be included in the study all participants had to be over 18 years and able to speak, read and write English. Additionally, injured persons had to have a current or previous claim related to musculoskeletal or psychological injury; employers had to be from medium to large sized businesses with more than 20 full time equivalent staff and one claim in the last 12 months, and GPs had to have had experience in treating injury compensation claimants.

GPs were purposively sampled by geographic location of their practice, years of experience as a GP and percentage of patients with WSV and/or TAC claims seen per year. Existing relationships between the research group and WSV and the TAC were leveraged to recruit compensation personnel and snowball techniques were used to recruit additional agents in a range of roles. Employers and injured people were recruited from an existing database of claims currently held by WSV.

### Data collection

Following written informed consent, interviews were conducted by two qualitatively-trained research assistants. Interviews were between 45 and 60 min and covered topics such as experiences in navigating health and compensation systems, opinions of what the GP’s role should be in RTW, the actual role GPs played in RTW and the barriers and enablers of RTW. Interviews were audio-recorded and transcribed verbatim.

### Data analysis

After transcription, data checking and data cleaning, the interviews were thematically analysed [26, 27]. Initial coding schemata were developed manually by four team members using inductive methods and then transcripts were coded and cross-checked to verify interpretation. Differences were resolved by consensus. After this process, the entire team met to confirm the final interpretation and to clarify the key study findings. Thereafter, transcripts were entered into NVivo 10 to facilitate data management and further analysis. The Monash University Human Research Ethics Committee approved the study.

## Results

Participants included GPs (18 male (m)/ 7 female (f); mean age = 52 years of age (yo)), employers (9 m/16 f, mean age = 45 yo), compensation agents (4 m/22f, mean age = 34 yo) and injured workers (11 m/6 f, mean age = 48 yo). Employers involved occupational health and safety staff, including RTW coordinators (*n* = 14), administrative staff, including human resources personnel (*n* = 7) and executive officers such as company directors (*n * = 2) (data were missing for two entries). There was diversity in providers’ mean years of experience (ye) in their current job role (GPs = 24 ye; employers = 9 ye; compensation agents = 7 ye) as well as in injured workers’ injury types, time since injury, RTW status, economic status, family size and nature of employment (see Table 1).

**Table 1:** Characteristics of injured workers

Mean Age (SD)	48 yrs (13.7)
Gender n (%)	Men = 12 (71)
	Women = 5 (29)
Primary injury type n (%)	Musculoskeletal = 12 (71)
	Psychological = 3 (18)
	Both = 2 (12)
Clients back at work n (%)	Yes =8 (47)
	No = 9 (53)
Time since injury n (%)	3 - 6 months = 5 (29)
	6 - 9 months = 0
	>9 months = 12 (71)
Weekly household net income n (%)	$300 - $600 = 2 (12)
	$600 - $900 = 8 (47)
	$900 - $1500 = 6 (35)
	>$1500 = 1 (6)
Weekly household expenses n (%)	<$660 = 6 (35)
	$660 - $1000 = 9 (53)
	$1200 - $1500 = 1 (6)
	>$1500 = 1 (6)
No. of dependents n (%)	None = 7 (41)
	One = 4 (24)
	Two = 4 (24)
	Three = 2 (12)
Nature of employment n (%)	Full-time = 16 (94)
	Part-time = 1 (6)

Five themes describing factors influencing GP certification were identified: 1. Divergent stakeholder views about the GP’s role in facilitating RTW; 2. Communication between the four stakeholder groups; 3. Conflict between the stakeholder groups; 4. Allegations of GPs and injured workers misusing the compensation system and 5. The layout and content of the certificate itself.

### Divergent views about the GP’s role

GPs, employers, compensation agents and injured workers recognised the benefits of RTW and considered GPs to be the main gatekeeper for facilitating injured workers’ RTW.

*Psychologically it’s important to get back into the workforce. I think that’s very important for the injured workers to know that they haven’t been cast off in the part quite yet (Injured Worker (IW) #12, m, 51yo).*

*If the GPs just encourage them to return to work, that they’ve got to get back, its better. It’s probably better for their mental health more than their physical health (Employer (EMP) #2, m, 64yo, 8 ye).*

*If a GP can focus on getting their patient back to what was normal in terms of the injury and return to work… it can help the patient in the long term from developing other secondary psychological issues or becoming demoralized at not being able to work (Compensation scheme agent (CS) #24, f, 49yo, 17 ye).*

*Work is an antidepressant. A person might be depressed because they can’t get out. At work they get to feel better and they feel that they can contribute again to society as before (GP#9, m, 65yo, 40ye).*

The various groups expressed divergent views about the GPs role in the certification process. GPs saw their role as primarily being an advocate for the patient; however, employers and compensation scheme agents thought that part of the GP advocacy role should also include promoting RTW

*I see that I’m an advocate for the patient and I’m also basically mostly trying to concentrate on treating their actual medical problem or the injury (GP#20, f, 47yo, 21 ye).*

*They [GPs] are the best advocates for the clients so therefore they need to have a strong discussion about it [RTW] … they really need to promote return to work. I just wish they could open their eyes a little bit and look at it (CS#14, f, 29yo, 3ye).*

Injured workers did not use the word ‘advocacy’ when describing their GP’s role. Instead, they said their GPs were ‘good,’ ‘supportive’ and ‘caring’ with their ‘finger on the pulse'. Activities they recalled GPs undertaking on their behalf included monitoring recovery progress, making appropriate referral, coordinating case management, helping workers navigate the health and compensation systems and, for some, eventually certifying RTW capacity. Workers valued continuity of care in their relationship with their GP and the feeling of being cared for. Only four workers felt that their GP had done little beyond complete paperwork:

*The GP, he’s a nice guy, he doesn’t tell you anything. He writes medical certificates for you but he doesn’t offer advice. I don’t know if it’s his role to do any of that? (IW#4, m, 48yo).*

Further analyses of these four cases revealed that the patients had reported either conflict between the GP and another provider (e.g. an independent medical examiner) or the belief that the GP did not have sufficient knowledge and training to address the injury. In two of the four cases, referrals to specialists had been made. However, prohibitive costs and long waiting times meant that injured workers relied predominantly on their GP to manage their recovery. For time-poor GPs working in busy environments where a multitude of issues presented, grappling with complex cases of injury, illness and recovery was difficult. Injured workers, employers and compensation agents recognised these challenges. One employer said, “I do understand they are crazy busy but it will be beneficial to us, to them and to the employee if they will get (sic) more contact [with us]” (EMP#10, f, 28yo, 1.5ye).

### Poor communication between the four stakeholders

GPs perception of themselves as patient advocates meant that they typically placed greater value in their relationships with their patients than they did in their dealings with compensation agents and employers. Certification by GPs was usually performed in consultation with the patient only, without consultation with employers and compensation agents. According to compensation agents and employers, relying solely on one source for all information and communication was problematic and could adversely influence certification and RTW outcomes.

*In my experience, GPs are really only certifying a worker to what the worker tells them that they can do. The GP has not gone out to the work site, the GP probably won’t have a good understanding of what a worker, what a worker’s role involves, whether they are a labourer or an office worker or everything in between, they wouldn’t have a good understanding of those duties. So at the end of the day … they have to trust what the worker says (CS#5, f, 24yo, 1ye).*

According to employers, a lack of GP knowledge and understanding of the injured worker’s job role and work environment often resulted in certificates that were unclear and with vague work restrictions.

*Every time I read them [certificate] it just says cannot lift over 5kgs … It doesn’t go into anything broad (EMP#9, 35yo, 10ye).*

In addition to prioritising the doctor-patient relationship, GPs reported other factors that influenced their limited interactions with employers and compensation agents: time constraints, lack of remuneration for the GP’s time spent on the phone with compensation agents, GPs’ frustration and lack of understanding of the compensation system, high paperwork burden and maintaining patient privacy.

*They [injured workers] have to go to court, they have to litigate and so on, which is annoying and then we have to go up there, go and testify for the patient. It’s an annoying process, (GP#9, m, 65yo, 40ye).*

For employers, there was broad consensus that GPs’ certification could adversely affect the finances of the business through increasing insurance premiums and compensation costs if workers were certified unfit for long periods of time. Additionally, putting some injured workers on light duties but paying them the same rate as those who performed more demanding work raised questions of fairness and fair remuneration in the workplace. Employers said that the easiest way to overcome these concerns was through improving communication with the GP:

*This is where the communication between the GP and us is critical because if he is unsure whether to send him back because he is unsure of what he does, then he will just give him another week and that costs everyone (EMP#3, 60yo, 10ye).*

### Conflict and GPs’ certification

Conflict appeared to be a major deterrent to certifying capacity. GPs reported conflict between their patients and ‘the system’ and feeling ‘stuck in the middle:’

*The heart-sink patients … are patients – the WorkCover ones – that make us all shudder. They’ve been on WorkCover forever, there’s all this conflict between the insurance company itself, the employer, the patient [and] we are stuck in the middle. You write report after report saying the same thing; it goes nowhere (GP#19, f, 37yo, 13ye).*

Many GPs said that heart-sink patients often wanted more time off despite being medically capable to RTW. Direct conflict arose when these patients’ views about RTW diverged significantly from the GPs clinical opinion. Managing such conflict, GPs said, required compromise by sometimes certifying patients as unfit for work for a short period of time:

*I try to assess how honest the person is and sometimes the sick note can be shortened. [Some patients] may believe they require one, two or three days off work [while others] for the same problem may require less time off work. We sometimes have to compromise as to how sick the person actually is (GP#3, m, 53 yo, 35 ye).*

GPs talked about the nature of patient’s pleas for more time off work, with one describing it as, “A tug on your heart strings” (GP#5, m, 36yo, 7ye).

Allegations (but no direct accounts) of GPs feeling ‘threatened’ and ‘intimidated’ by patients were mentioned by GPs:

*I think some patients can be quite threatening. I don’t envy some GPs… I mean you’ve got remember 25 % of GPs are assaulted in their career and the reason I now work in (name of a reasonably affluent suburb) is because the health outcomes are better (GP#4, m, 58yo, 32ye).*

Indirect conflict arose when GPs were not directly embroiled in a disagreement with other parties (such as compensation authorities or employers) but their injured patients were. In these cases, GPs reported feeling more empathetic towards patients because of their strong advocacy role and their privileging of the doctor-patient relationship

*Basically it’s for us (GPs) to try and help the worker as the injured person, do the right amount of work and the right kinds of tasks, and the things in the return to work process. And not do too much or too long. And it’s the boss’s job to try and… I mean it’s a tug of war sort of thing in a sense isn’t it? You can see the patient is kind of [in] the middle of that tug of war and we have to try and help (GP#25, m, 50yo, 25ye).*

### Allegations: Patients and providers misusing the system

Perhaps because of their perceived role as patient advocates, compensation agents reported that GPs were more likely to certify incapacity to work when the patient was experiencing extenuating social circumstances such as lack of available childcare or marital discord.

*We have clients who have disabled children and they weren’t caring for them primarily prior to the accident. This is a perfect opportunity to getting benefit to the income replacement while they are unfit. So that can cause a GP to also have that human side and say, “Jeez this would really help them [family]. At the scheme they are able to continue to support that so I will help them out by writing they are unfit (CS#12, m, 27yo, 3ye).*

GPs were aware of the fact that the health and compensation systems did have vulnerable points, which some doctors and patients did exploit. For example, there were assertions by GPs and compensation agents of ‘doctor-shopping,’ i.e., patients who went to multiple providers until they obtained the certificate of in/capacity, which they wanted. Related to this practice were allegations of doctors who certified for as much time as the injured worker wanted. Such GPs were purported to neither weigh the medical evidence nor circumstances of the claim and were pejoratively termed ‘how long’ doctors by GPs and compensation agents.

*I think that people gravitate towards the doctors that are going to give them what they want … There are clear WorkCover doctors, perhaps inappropriately but often called ‘Dr How Long’ by the agents and, ‘How long do you want your certificate for?’ That’s where the phrase came from (CS#26, f, 41yo, 14ye).*

*Most of them [don’t] go back to their own GP. They go looking to find ‘Dr How Long’ who gives them all the time off that they want (GP#16, f, 51yo, 22ye).*

It is important to emphasise that in our sample, neither GPs nor injured workers identified as being either indiscriminate certifiers or doctor-shoppers. In fact, when asked, all participants said that fraudulent claims were rare.

*I also accept that there are a number of patients who commit fraud on insurance companies and there are a number of patients who are malingerers, but malingering is very uncommon in my experience (GP#10, m, 66yo, 38ye).*

### Issues concerning the layout and content of certificates

All four groups complained about the layout and content of the certificate (see Additional files 1 and 2. Certificates for Assessing Capacity). GPs said that they did not “understand what the writer of the form wanted from them” while compensation scheme agents and employers complained that the certificate of capacity needed to promote a stronger RTW focus by providing an option for GPs to state what activities the injured worker could do rather than what they could not do. Compensation scheme agents and employers said the certificate made it easier to certify workers as ‘unfit’ rather than recommend alternate or modified duties as the latter required lengthier and more detailed clinical consultations.

*The certificate makes it easy to certify total incapacity, to certify someone as totally incapacitated for work you only have to write two dates on the certificate – ‘from’ and ‘to’(CS #18, m, 61yo, 4ye).*

The need to provide more options on the forms, which GPs and specialists could then ‘tick and flick,’ was also mentioned by compensation agents. Providing prompts allowed more information to be gathered about capacity; for example, how long an injured worker could sit or stand or how much he/she could lift. Compensation agents said this information could contribute to a RTW plan.

*In terms of doctors and surgeons and treaters (sic) I find that their time is very minimal so I have so many tick and flick faxes, which I have literally jotted it out for them and all they have got to do is tick a couple of boxes and write a couple of numbers and so forth (CS#6, f, 27yo, 8ye).*

Similarly, GPs valued the prospect of having a more instructive pro forma:

*There is just a box for us when we want someone to go back with modified duties. There is just an empty box and we are supposed to give this complete answer as to what a whole person is capable of doing. Maybe if there was more of a pro forma type of thing (GP#21, f, 39yo, 7ye).*

Finally, all four groups wanted certificates to be electronically completed and integrated into online and real-time systems to enable consistent and coordinated communication, which would facilitate provider uptake.

*The new doctor I’ve got has got it on the computer and it’s the same sort of form … I’m just wondering if … it could be send electronically to [insurer] directly wouldn’t that save time and energy, you know costs? (IW#7, m, 46yo).*

## Discussion

Our study presents the first qualitative data on GP certification practices in Australia [also see 5, 28]. Drawing on a unique sample of GPs, compensation agents, injured workers and employers, our findings show certification is an administrative and clinical task underpinned by a host of social and systemic factors. These social and systemic factors are inextricably linked. For example, because GPs saw themselves as the patient advocate they were empathetic of their patient’s circumstances and avoided patient conflict, sometimes certifying fitness incapacity based on social (e.g. childcare difficulties, marital discord) rather than clinical factors. As a consequence of this role, GPs also valued patient communication above communication with other stakeholders, which influenced their certification practices, which in turn influenced patients RTW outcomes, productivity for employers and costs for compensation schemes (the latter two are structural factors). Similarly, structural factors, such as the layout of the certificate, its lack of instruction, absence of electronic and online integration also delayed effective communication between GP’s and compensation agents and employers. Structural factors such as the administrative requirements placed on GPs by compensation schemes also related to GP’s social perceptions that they were unfairly remunerated for the amount of paperwork they were required to complete.

These findings are similar to studies that have investigated GP certification practice in other countries. For example, UK studies show that when GPs encounter complex, chronic, or ambiguous cases, they find themselves unwilling intermediaries between their injured patients and compensation authorities, moderating access to a system about which they had little knowledge [29]. GPs expressed difficulties occupying this gatekeeper role and at the same time acting as a patient advocate. Seminal work by Hussey et al., from Scotland, found that most GPs privileged their relationship with their patients over their relationship with government institutions to the extent that they occasionally misused the system, often citing reasons such as patient confidentiality, stress, time-shortages, avoiding conflict with the patients and disillusionment with the system, for doing so [29]. Similar to the findings of this study in an Australian context, GPs’ certification in other contexts has also been found to be heavily influenced by the patient’s social circumstances [30–32]. For example, a Swedish study showed that ‘sick-listing’ has become a solution to many non-medical problems such as family conflict and social problems [32].

Conflict is a recurring theme in qualitative investigations of GP certification. Conflict arising from patient demands for sickness certification that contradict the GPs medical opinion has been reported in studies from Sweden and the UK [32–34]. Conflict arising from difficulties negotiating with compensation authorities and employers has also been reported in Northern Europe [35]. In the UK, conflict has been suggested to be one reason why some GPs prefer to have no role in sickness certification [33, 34].

The consistencies between the current findings and those of international studies with different compensation systems, reinforce the complexities of certification and RTW.

### Implications for policy and practice

To try and overcome some of these barriers, the UK Government encouraged GPs to issue injured workers with ‘fit notes’ rather than ‘sick notes’ to emphasise what people can do in the workplace and, thereby, facilitating labour market retention [7]. Several studies have since shown that there is recognition among UK GPs about the health benefits of work and that the fit note functions as a prompt to facilitate conversations around an earlier return to work [36–38]. The fit note also enables discussion between employees, employers, and GPs about appropriate, timely RTW and reduced sickness absence, which has wider benefits to injured workers, employers, and the economy [39]. However, there are concerns that the fit note has increased consultation times [38] and that GPs are not completing the fit note as intended [40]. Training GPs on the correct completion of fit notes is critical to facilitating change in current practice [7, 40].

In this study in the Australian context, the health benefits of RTW and the central role that GPs play in the process were recognised by the different stakeholders. The next stage is to take advantage of those targets that are easily achievable (e.g. guideline development) so as to make positive changes in GP sickness certification in Australia.

A first step in overcoming barriers identified in this study may be clarifying the GPs’ role in RTW. This need not delineate between GP as patient advocate versus GP as RTW certifier. Rather, by leveraging the strong advocate role that GPs feel towards their patients, evidence-based education could be provided to GPs to communicate directly to their patients about the benefits of early RTW. Similarly, training GPs, through continuing medical education and medical students in medical schools, about RTW, workers compensation systems and how to deal with conflict could help reduce the number of ‘unfit’ sickness certificates currently being issued as would trialling better ways to streamline communication between stakeholders and developing guidelines around sickness certification.

Importantly, any proposed intervention needs to be supplemented with training to sustain changes in certification practice. UK data on practice change show that behaviour change is limited when GPs are neither aware of the guidelines nor trained about sickness certification [15, 40]. Our data demonstrate that Australian GPs want more clarity around current certification and more guidance on how to certify. Yet to date, there are no guidelines on this subject. This is ‘low hanging fruit’, which could be easily addressed in the short-term through research and rigorous guideline development and, if carefully implemented, in the medium to long-term, could reap many benefits.

Concomitantly, Australian compensation agents and employers want to see a shift in the certificate from a focus on incapacity to one on capacity, much like the UK ‘fit note’ [7]. One way to achieve this is to put in place levers that encourage GPs to think about capacity through certification. Promisingly, the local compensation authorities are in the process of redesigning the certificate; however, the new iteration and its impact remain to be seen.

To the best of our knowledge, ours is the first study in Australia and internationally to explore various stakeholder perspectives on GPs and RTW. A diverse and large qualitative sample size and a careful approach to data collection and analysis give reliability to our findings. Our work is limited by the exclusion of other stakeholder perspectives such as lawyers, medical specialists, occupational health practitioners and allied health professionals. In addition the sampling strategy employed in this study restricts the generalizability of the study findings. For example, it is possible that stakeholders with negative experiences of GP certification saw the study as a chance to have their voice represented. In this way stakeholders with more positive experiences may have been under-represented. However, the comparability of our results to international literature adds validity to our work. Finally, whilst this study was conducted in Victoria, Australia, similar compensation schemes exist in other states in Australia and therefore these findings may generalise to the wider Australian context.

## Conclusion

The results of this qualitative study highlight the value of exploring GP sickness certification from the perspectives of all key stakeholders. Drawing on a unique sample of GPs, compensation agents, injured workers and employers, a number of social and systematic factors were found to influence GP sickness certification. The results highlight potential opportunities such as practice guideline development and improvements to the certificate itself that may be targeted to improve GP sickness certification, RTW outcomes and, ultimately, the health of injured patients.

## Additional files

Additional file 1:
**TAC Certificate of Capacity.** (PDF 55 kb) Additional file 2:
**WSV Certificate of Capacity.** (PNG 190 kb)

## Abbreviations

GPs: General practitioners
RTW: Return to work
UK: United Kingdom
WSV: Work Safe Victoria
TAC: Transport Accident Commission
ye: Years of experience
yo: Years of age
m: Male
f: Female
IW: Injured worker
EMP: Employer
CS: Compensation scheme agent
