# Supplementary material for: General practitioners and sickness certification for injury in Australia
Source: BMC Fam Pract. 2015 Aug 15;16:100. doi: 10.1186/s12875-015-0307-9 (PMC4537596; doi:10.1186/s12875-015-0307-9)
Supplement: Additional file 1: — TAC Certificate of Capacity. (PDF 55 kb) [file 12875_2015_307_MOESM1_ESM.pdf]

MEDICAL CERTIFICATE: INITIAL

Privacy

The TAC will retain the information provided and may use or disclose it to make further inquiries or assist in the ongoing management of the claim or any claim for common law damages. The TAC may also be required by law to disclose this information.

Without this information, the TAC may be unable to determine entitlements or assess whether treatment is reasonable and may not be able to approve further benefits and treatment. If you require further information about our privacy policy, please call the TAC on 1300 654 329 or visit our website at www.tac.vic.gov.au

Patient details

Name

Address

Post Code

Claim number

Date of birth / /

Date of accident / /

Date examined / /

Date first examined / /

Current clinical diagnosis

When examined the patient was found to have the following accident-related conditions. *Attach additional notes if required*

Condition	Left	Right
1.		
2.		
3.		
4.		
5.		

Are these conditions consistent with the patient's description of the cause?

☐ Yes ☐ Uncertain

Did the client receive a head injury? If so, please state scores for:

- GCS _____ PTA (days) _____

Please indicate sites of pain on this body map

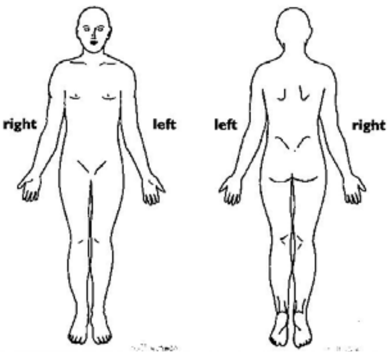

Relevant past medical history as known

Are you aware of any pre-accident injury, illness or condition that may impact on the treatment of, or recovery from, the injuries incurred in the transport accident?

☐ Yes ☐ No

If yes, provide relevant clinical details of injury illness or condition

Does the patient require any support services?

Please detail the type of support services the patient requires, e.g. housekeeping, child minding, taxi transport. Include the frequency, e.g. number of days per week and hours per day required

Support service	Frequency	Until
1.		
2.		
3.		
4.		
5.		

Briefly explain how the need for the support services relates to the transport accident injury(s) and the patient's functional status in the comments section below. *Attach additional notes if required*

Capacity for work *specify reason and restrictions in comments section below*

	From	To
Expected to be fit for usual duties		
Fit for modified/alternative duties		
Unfit for any work duties and has no work capacity		

Comments *e.g. work restrictions, work capacity, need for support services*

Provider details

I certify that I have clinically examined this patient. The information and medical opinions contained in this certificate are, to the best of my knowledge, true and correct.

Provider name, address and phone no. *Use practice stamp where possible*

Signature

Days/hours available

Date / /

Please attach any information that may be relevant.

TRANSPORT ACCIDENT COMMISSION

60 Brougham Street
GEELONG VIC 3220
GPO Box 2751
Melbourne VIC 3001
DX 216079 Geelong

Telephone 1300 654 329
STD Toll Free 1800 332 556
Facsimile 03 9656 9323
www.tac.vic.gov.au
ABN 22 033 947 623
Facsimile 03 9656 9323

State Government Victoria
