# Supplementary figures and images for: General practitioners and sickness certification for injury in Australia
Source: BMC Fam Pract. 2015 Aug 15;16:100. doi: 10.1186/s12875-015-0307-9 (PMC4537596; doi:10.1186/s12875-015-0307-9)

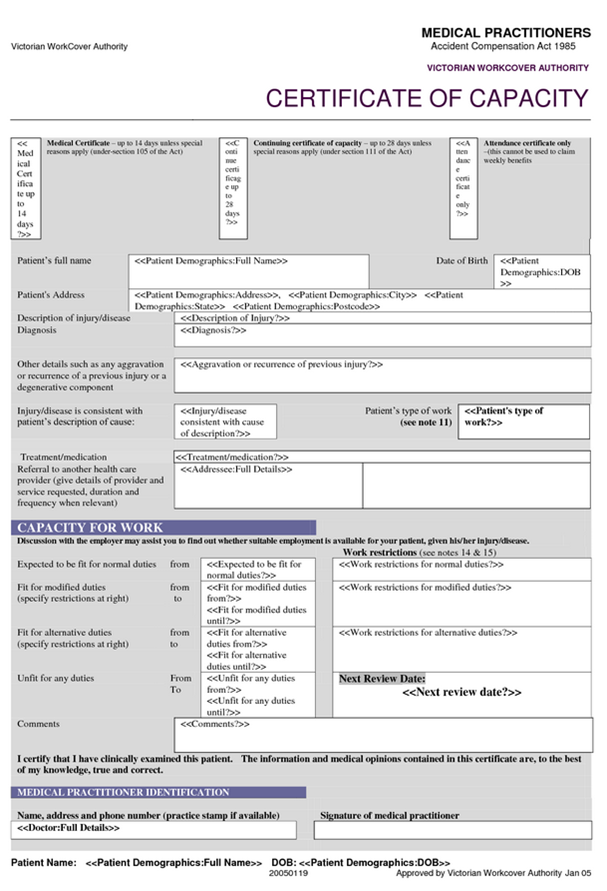

Supplement: Additional file 2: — WSV Certificate of Capacity. (PNG 190 kb) [file 12875_2015_307_MOESM2_ESM.png]
